# Assessment of Insomnia Symptoms, Quality of Life, Daytime Sleepiness, and Psychoactive Substance Use among Polish Students: A Cross-Sectional Online Survey for Years 2016–2021 before and during COVID-19 Pandemic

**DOI:** 10.3390/jcm11082106

**Published:** 2022-04-09

**Authors:** Mateusz Babicki, Patryk Piotrowski, Agnieszka Mastalerz-Migas

**Affiliations:** 1 Department of Family Medicine, Wroclaw Medical University, 51-141 Wroclaw, Poland; ma.babicki@gmail.com (M.B.); agnieszka.mastalerz-migas@umw.edu.pl (A.M.-M.); 2 Section of Epidemiology and Social Psychiatry, Department and Clinic of Psychiatry, Wroclaw Medical University, 50-367 Wroclaw, Poland; 3 Division of Consultation Psychiatry and Neuroscience, Wroclaw Medical University, 50-367 Wroclaw, Poland

**Keywords:** sleep disorders, insomnia, quality of life, students, young adults, COVID-19

## Abstract

Sleep disorders are a serious health problem worldwide, and insomnia is their most common manifestation. An increasing number of people have insomnia every year, young adults, especially. Due to the importance that sleep has in almost every aspect of our lives, the need to monitor disturbances in circadian rhythms has arisen. Therefore, this study aimed to assess the prevalence of sleep disorders among Polish students, including their quality of life (QOL) and drug use patterns. The study also investigated associations between sleep, QoL, and drug use. The study was conducted in 2016–2021 based on the self-made sociodemographic questionnaire, as well as standardized psychometric tools: Athens Insomnia Scale (AIS), Epworth Sleepiness Scale (ESS), and Manchester Short Assessment of quality of life (MANSA). A total of 14,844 students participated in the study. The majority were women (80.7%), of which 3425 (23.1%) were medical students, with the most numerous representing medical and dental faculties, 1884 (57.2%). Before the COVID-19 pandemic, 52.1% of the surveys were collected; 54.1% of respondents had insomnia as indicated by the AIS scores, and 26.1% displayed sleepiness during the day. Female students, the first-year college students, more often suffered from sleep disorders. Drug use was widespread among Polish students, correlating with sleep assessment results and QoL. In conclusion, sleep disorders are a significant clinical problem among Polish students. Female and junior students’ years are more prone to sleep deprivation. Insufficient sleep can be associated with a lower QoL score and psychoactive substance use. The effects of the COVID-19 pandemic on sleep are not conclusive, because there was decreasing quality for longer sleep durations. In order to analyze these associations, there is a need for further in-depth study.

## 1. Introduction

Sleep disorders are a significant health problem worldwide, and insomnia is the most common disease that affects increasingly more people every year [1,2]. Young adults, often students, are more prone to sleep deprivation [3]. Several reports have estimated that even 40–60% of young adults have sleep deficits [4,5,6,7,8]. Biological and social factors and incorrect behavior patterns are primary reasons for such phenomena. Because of their age, the functioning of most students can follow symptoms of the so-called “youthful” delayed sleep-wake phase disorder. Namely, their evening and night periods become preferential to increased activity, while the day, especially in the morning, is a period of sleep and rest. Getting up early, necessary to fulfill new adult social roles, is very difficult, and the amount of sleep becomes insufficient [9]. Undoubtedly, the changes in social life functioning have a significant impact. A period of education at the university level is associated with many new life experiences such as changing the place of residence, increased independence, changing peer groups, and new duties related to educational learning [10]. In addition, the population of students rarely follows proper sleep hygiene and frequently overuse psychoactive substances affecting sleep physiology [11,12,13].

Sleep is fundamental to life functions. Healthy sleep is necessary to maintain mental and physical well-being and proper emotional and social functioning, while its disturbances may have many adverse outcomes [14,15]. First, sleep deficit may lead to excessive daytime sleepiness [10]. Second, individuals suffering from sleep disorders exhibit problems with attention, memory, and concentration, which result in poor performance in study and work [16,17,18,19]. The university student population is also predisposed to chronic diseases such as diabetes, hypertension, obesity, or myocardial infarction with an increased risk of death [20,21]. These people have a lowered quality of life (QoL) and an increased risk of developing psychiatric diseases, and a tendency to risk behaviors and poor social relations [22]. Because sleep deterioration enormously affects mental health, physical health, and everyday functioning, there is a need of close monitoring of the COVID-19 pandemic deteriorating almost every aspect of human life, as shown by recent studies [22]. Moreover, the very recent studies have indicated that the adverse psychological outcomes of the COVID-19 pandemic such as anxiety, depression, sleep disorders, and post-traumatic stress disorder more often affect younger adults, including university students [23]. There is also evidence of changes in the psychoactive drug use patterns in the COVID-19 pandemic, depending on the data collection period, sample population, and the initial level of drug use [24,25].

At the beginning of the 1970s, the definition of quality of life (QOL) was introduced to medicine. Since then, there has been growing interest in applying QoL into medical practice. According to the WHO definition, QoL is a subjective assessment of an individual’s life situation concerning the individual’s culture, value system, goals, expectations, and interests [26]. Therefore, measuring a patient’s QOL provides an essential possibility for assessing a patient’s perspective concerning an individual life situation. As shown, patients’ subjective feelings influence, to a large extent, their behavior. Several studies have shown that patients with decreased QoL due to a specific therapeutic therapy are more likely to give it up despite clinical effectiveness. Nonetheless, the nature of QOL measurement is challenging. Current research usually uses a questionnaire technique, covering many aspects of life and being interpreted by having a total score value. An exemplary survey measurement is the Manchester Short Assessment of quality of life (MANSA) [27,28,29].

To sum up, studies so far have shown that there is a close correlation between insomnia and daytime sleepiness, with people suffering from insomnia having a higher level of daytime sleepiness. Additionally, both of these parameters significantly lower the assessment of the quality of life of students. Moreover, it has been proven that both women, students of younger years and students of medical faculties show greater problems with sleep than others [2,6,10,14,15]. The negative impact of the ongoing COVID-19 pandemic on sleep quality and the assessment of quality of life has also been proven. During the ongoing pandemic, an increase in sleep problems was observed both in the general population and among students [22,23,24,25]. Moreover, the period of the pandemic also contributed to changes in the scope of stimulants used, with the impact varying depending on the period of data collection and the study group. Due to the connections between sleepiness, insomnia, quality of life, and the drugs used, and the impact of the COVID-19 pandemic on them, we decided to conduct a study that combines individual aspects [24,25].

This study aimed to assess disorders of circadian rhythms among Polish students, including their QoL and psychoactive drug use patterns. In addition, is there a relationship between insomnia, daytime sleepiness, and quality of life? Does the use of psychoactive substances affect the level of insomnia, daytime sleepiness, and the assessment of the quality of life, and is there a difference in their assessment in terms of gender, field of study, and year of study? Finally, has the COVID-19 pandemic contributed to the deterioration in the quality of sleep and life of Polish students? Based on previous reports [1,2,4,20,21,22,23,24,25], the authors formulated the following research hypotheses: (1) Sleep disorders are common among Polish students. (2) Sleep disorders would be higher among female university students. (3) There would be an association between sleep disturbance and QoL. (4) The COVID-19 pandemic would exacerbate sleep disorders and reduce QOL among students. (5) Male university students would be more prone to risk behaviors, including the use of psychoactive substances.

## 2. Materials and Methods

### 2.1. Methodology

The study used a survey questionnaire design using standardized questionnaires distributed online via the Facebook social network in groups associating students. The students’ status in most cases was confirmed via their membership. The survey was administered to students living and studying in Poland, and participation in the survey was anonymous and voluntary. The survey data collection covered the period from 31 January 2016 to 1 January 2021.

At each stage of the study, the respondents had the opportunity to terminate their participation without giving any reason. After being familiarized with objectives and the research methodology, the respondents were asked to give informed consent to participate in the study. Consented participants were then requested to continue the study procedure. The survey questionnaire consisted of two sections. The first one assessed socio-demographic status of the respondents, including age, sex, place of residence, field of study, and year of study. Then, the survey assessed frequencies of using psychoactive substances (alcohol, cannabinoids, psychostimulants) and hypnotics. The second part involved three standardized psychometric tools: Athens Insomnia Scale (AIS), Epworth Sleepiness Scale (ESS), and Manchester Short Assessment of quality of life (MANSA).

#### 2.1.1. Athens Insomnia Scale (AIS)

The AIS scale is an 8-item tool for quantitative evaluation of insomnia symptoms based on the ICD-10 criteria. It uses a four-point Likert scale (0—no difficulty, 3—severe difficulty) assessing difficulty in falling asleep, waking up at night, waking up in the morning, total sleep time, sleep quality, well-being the next day, mental and physical fitness the next day, and sleepiness during the day, occurring at least 3 times in the last 14 days. The maximum score that can be earned is 24. The Polish adaptation of AIS was used in which the cut-off point for diagnosing insomnia was set at 8 scores. The measurement characterizes high sensitivity (93%) and specificity (85%). Moreover, a high reliability of the Cronbach’s alpha was shown, yielding a value of 0.832 [30,31,32].

#### 2.1.2. Epworth Sleepiness Scale (ESS)

The ESS scale measures excessive sleepiness and is one of the most common in making the diagnosis of sleepiness. The ESS consists of 8 questions about the likelihood of falling asleep in 8 typical everyday situations (e.g., watching TV, sitting in a public place, lying down and relaxing in the afternoon, etc.). The maximum ESS score is 24. The following point criteria were adopted in the present study: 0–10 points means no daytime sleepiness, 11–14 points—sleepiness, and 15 points and above—pathological sleepiness, which requires consultation with a doctor. The reliability of the scale measured with Cronbach’s alpha coefficient was 0.755 [33,34,35].

#### 2.1.3. Manchester Short Assessment (MANSA)

The MANSA measures the subjective assessment of several aspects of an individual’s life satisfaction. MANSA consists of 16 questions covering individual spheres of life. In 12 questions, the respondents give answers on a Likert scale from 1 (it could not be worse) to 7 (it could not be better). The remaining 4 items include affirmative (2 points) or negative (1 point) answers. The maximum MANSA score is 92, and the QoL interpretation involves the total scored obtained by respondent, where the higher the value, the better the assessed QoL. Some reports may also use individual questions to evaluate life satisfaction. Cronbach’s alpha was 0.810 [36,37].

### 2.2. Statistical Analysis

The analysis employed numerical and rank-based values. The chi-square test was used to assess significant differences in the demographics of the university student population before and during the pandemic and the frequency of drug use in both periods of time. The age differences between groups before and during the pandemic was tested using the *t*-test. Linear regression examined the effects of sociodemographic variables and stimulants on the AIS, ESS, and MANSA scores. The linear regression models included two predictors, including their interaction: the first predictor was “Pandemic” (with two levels: “Before” vs. “During” Pandemic). Separate models included gender (female vs. male), year of study (I–VI), major (medicine, engineering, humanities, economics, biology), type of faculty (medical studies), alcohol consumption (yes/no), consumption of cannabinoids (yes/no), psychostimulants (yes/no), and use of hypnotics (yes/no). The effects of the period of the study (“before” and “during” a pandemic) and demographics on MANSA life satisfaction were analyzed using the ordinate polynomial logistic regression. It included dependent variables such as the subsequent MANSA items with rank values and the predictors of the study period and demographic variables in separate models. The relationships between drug use and the pandemic and demographic variables were investigated similarly. The correlation between the scales was performed using Pearson’s correlation coefficient. The Statistica program (13.3.) was used to compute *t*-test and chi-square statistics and general linear models. The ordinate polynomial logistic regression models were built with the MASS package in the R 4.0.4 environment.

## 3. Results

### 3.1. Characteristics of the Respondents

Detailed characteristics of the population are presented in Table 1. A total of 14,844 university students in Poland with an average age of 21.82 ± 2.8 participated in the study. A total of 7735 (52.1%) surveys were collected in the period before the declaration of the COVID-19 pandemic. The majority were women, 11,985 (80.7%), and 5,352 humanities students (36.1%). Similar frequencies were observed both before and during the pandemic. The study included a group of 3425 medical students (23.1%), in which the most numerous group were students from the medical and medical-dental faculty, 1884 (57.2%). The study was conducted according to the Helsinki Declaration and was approved by the Bioethics Committee at the Wroclaw Medical University.

### 3.2. Sleep Disturbance

Detailed results of sleep disturbance are presented in Table 2. Polish students obtained a mean AIS score of 8.40 ± 4.35, and as many as 8041 (54.1%) students scored at least 8 points, showing insomnia [32]. A total of 3644 (24.5%) respondents said their sleep time was satisfactory, while 4120 (27.7%) had satisfactory sleep quality. Only 955 (6.4%) did not report daytime sleepiness. Specific single AIS measures for the entire study group and the COVID-19 pandemic are presented in Table S1.

For the ESS’s somnolence subscale, the mean score was 7.77 ± 4.19. Somnolence was diagnosed in 3872 (26.1%) students, of which 892 (6.1%) declared suffering from pathological somnolence. The most common predisposition of sleep is lying down and resting after lunch (91.1%). Nearly 5.8% of students admitted that there are situations when they could fall asleep while driving a car for a few minutes to stop in a traffic jam. The list of ESS items is presented in Table S2, which is part of the Supplementary Materials.

The analysis of sociodemographic variables indicated that women had significantly higher mean AIS and ESS scores. Moreover, higher AIS and ESS scores were observed among first-year students. Interestingly, medical students with the lowest AIS values had at the same time the highest ESS measure. There was a correlation between the stimulants and ESS measures of sleep. Higher ESS scores were associated with psychostimulants, cannabinoids, and hypnotics use. On the other hand, both psychostimulants and hypnotics affected the AIS measures for students having on average higher values in both scales. There was an inverse correlation for the effects of alcohol on sleep because individuals who consumed alcohol obtained lower results on average on both scales.

There was no significant difference in the AIS measures between the period before and during the pandemic, *p* = 0.061. The analysis of separate items showed a significant decrease in sleep qualities for both stages as sleep was rated as satisfactory by 30.09% of students before the pandemic as compared to a percentage of 25.20% after the declaration of the pandemic (*p* < 0.001). However, after the pandemic had started, the percentage of students with adequate sleep time increased (20.97% vs. 28.44%, *p* < 0.001). A detailed list is presented in Tables S1 and S2.

The analysis of separate items showed that the factors of the well-being of the next day (r = 0.702, *p* < 0.001) and the subjective assessment of sleep quality (r = 0.663, *p* < 0.001) impacted the final AIS score most.

Similarly, the higher ESS scores correlated with the probability of falling asleep while reading (r = 0.585, *p* < 0.001) and during rest after lunch (r = 0.579, *p* < 0.001).

### 3.3. Quality of Life Assessment

Detailed MANSA results are presented in Table 3. The study found that the mean QoL was 60.95 ± 11.25. Women assessed their QOL significantly lower than men (*p* = 0.043). Among the stimulants used, both alcohol and cannabinoids were associated with a higher QOL score, unlike with hypnotics and psychostimulants. The period of the COVID-19 pandemic significantly lowered the QoL among Polish students (*p* < 0.001). Moreover, people suffering from insomnia and daytime sleepiness showed a much lower subjective assessment of the quality of life (*p* < 0.001).

The analysis of separate MANSA scores showed that Polish students gave the highest ratings for their satisfaction with the sense of security and relationships with their roommates. Because of the ongoing pandemic, there was a significant decrease in the subjective assessment of mental health (OR 1.49, *p* < 0.001), as well as the general feeling of satisfaction with life (OR 1.3, *p* < 0.001). During the pandemic, Polish students gave higher ratings for satisfaction with the housing situation (OR 0.81, *p* < 0.001) and relations with roommates (OR 0.88, *p* < 0.001). A detailed list of the MANSA items and their relationship with the COVID-19 pandemic is presented in Table 4.

### 3.4. Correlations between Individual Scales

There were significant correlations between the AIS, ESS, and QoL measurements used in the study. It was shown that the increase in AIS values was positively associated with the mean ESS score (r = 0.223, *t* = 27.983, *p* < 0.001). However, there was an inverse correlation between the assessment of sleep and QOL, because higher AIS (r = −0.477, *t* = −66.204, *p* < 0.001) and ESS (r = −0.129, *t* = −15.923, *p* < 0.001) scores correlated with a lower subjective QOL assessment (MANSA).

### 3.5. The Pattern of Stimulants/Alcohol and Drug Use among Polish Students

The drug use related to stimulants is presented in Table 1. Detailed effects of sociodemographics and the pandemic on drug use are shown in Table 5. The most common stimulants were alcohol, consumed by 87.7% of respondents, but after the COVID-19 pandemic burst, alcohol use had decreased from 91.3% to 84%. Hypnotics were used by 13% of the respondents, and psychostimulants were used by 2.9% of the university/undergraduate student population. There was a significant influence of gender on the type of stimulants. Women used sedatives/sleeping pills more often, whereas men consumed alcohol, and used cannabinoids and psychostimulants more often. With alcohol consumption, we observed a distinct pattern that a higher year of study was associated with increased alcohol use. It was also shown that after the COVID-19 pandemic burst, the frequency of using hypnotics/sedatives by Polish students increased significantly (OR 0.76, *p* < 0.001). However, as the pandemic continued, alcohol consumption decreased (OR 1.35, *p* < 0.001).

## 4. Discussion

Sleep disorders are a significant health problem, not only among the elderly but now also among young adults. Therefore, the central aim of this study was to assess the prevalence of sleep disorders among university students, including their QoL and patterns of psychoactive substance use. In addition, the study determined how the COVID-19 pandemic affected students’ quality of life. The study proved a considerable health problem of Polish students because it showed that 54.1% of the population sample had symptoms of insomnia as indicated on the AIS scale [31]. To the best of the authors’ knowledge, this has been the first report on such a large group of Polish students, and it includes observations both before and during the COVID-19 pandemic, which makes the study strong and innovative.

The ESS results showed the occurrence of daytime sleepiness in 26.1% of students, of which as much as 6.1% should suspect pathological sleepiness. The present results are consistent with other reports indicating a significant increase in sleep problems among young adults. For instance, in a study conducted in Morocco, the prevalence of insomnia was found in 54% of the students’ population, and 9.4% of them suffered from daytime sleepiness. In another study on Hungarian students, 23% of the population had insomnia [38,39]. However, as previously observed by Ohayon [40], the prevalence of sleep disorders varies depending on the data collection period, population sample, and research methods. For example, a Polish study on the assessment of sleep disorders among 2413 young Poles indicated that 50.5% of them declared difficulties with sleep [41]. Another study by Kałduńska et al. [42] in 2019 based on standardized psychometric tools found sleep disorders in 28.3% of respondents, among whom there was a large number of students from universities in Krakow—19.7% [42,43]. However, in a Polish study on 1,649 students during the lockdown in Poland, insomnia was diagnosed in 42.03% of the population sample [44]. Interestingly, in a large population study by Kiejna et al. [2], the occurrence of sleep disorders among Poles over 15 years of age was estimated at 27.3%. As mentioned earlier, these discrepancies more likely depended on the different periods of data collection, various research methods, and the selection of the population sample.

In this study and many previous reports, it was observed that women much more often suffer from sleep disorders [2,6,39,41,45]. This report found that women scored higher in both AIS and ESS scales. These findings are not clear. Undoubtedly, women show a higher level of emotional excitability and therefore are more at risk of developing psychiatric diseases, including anxiety and depression, which are associated with sleep problems. It is believed that this results from sex steroid hormones whose levels are different between genders after puberty [46]. The concentration of hormones in women is related to the menstrual cycle, affecting assessments of the quality and duration of sleep [47,48]. Some studies have shown that high estradiol and progesterone levels were associated with prolonged arousal and wakefulness [49]. There was also a unidirectional growing intensification of both insomnia and daytime sleepiness in the first-year students, obtaining higher AIS and ESS scores. These effects may be explained by appearances of life changes associated with the university enrolment, including change of place of residence, new social roles, and new life obligations. These changes can induce anxiety and fear, correlated with the risk of developing sleep disorders, as confirmed in earlier reports [50,51].

Contrary to the previous observations, in this study, medical students showed significantly lower AIS scores than students from other studies. In ESS, these values were highest, and the percentage of people with a positive result was 30.24%. In a survey among medical university students, daytime sleepiness occurred in 35% of Malaysian students, 30.6% of Indian students, and 46.5% of Brazilian medical adepts [15,52,53]. It is believed that high sleepiness among medical students is associated with many hours of classes, study, clinical practice, often including night duty, and significant stress and mental strain [54].

The comparison of separate AIS items showed a moderate correlation between the result and the question about sleep quality (r = 0.663, *p* < 0.001). This effect seems to be consistent with the reports worldwide indicating that the quality, but not the time, of sleep is most important (r = 0.578, *p* < 0.001). This may also be the result of no AIS differences between periods before and during the COVID-19 pandemic. During the lockdown, universities were closed, and students switched to remote learning. Their scope of decreased duties could contribute to changing their sleep pattern. For instance, Gupta et al. [55] observed that students went to sleep late more often during the lockdown, got up later, and had a greater number of naps during the day, which reduced the quality of sleep.

Another explanation could be that students had longer sleep during the day because of life changes. Here, we observed that the percentage of students with normal sleep time increased from 20.97% to 28.44%, *p* < 0.001, while sleep satisfaction decreased from 30.09% to 25.20%, *p* < 0.001. It is clear that delayed falling asleep can reduce the amount of slow-wave sleep, significantly deteriorating its quality and exacerbating sleep problems [53,54]. Similar findings were also observed among Spanish students as there was an increase in sleep time in the initial period of the pandemic [25]. In contrast, in a study conducted in Jordan, 94.9% of students declared that the pandemic had affected their sleep habits, and 74% of them reported a subjective deterioration in sleep quality. Additionally, 71.3% of students showed symptoms of depression [22]. The COVID-19 pandemic is an unusual situation, having a considerable impact on people’s health and psychological condition. Recent reports have shown an increased risk of depression, anxiety, sleep disorders, and PTSD in almost every age group [23]. In addition, the present results of the subjective assessment of mental condition showed a decreasing tendency when comparing periods during and before the pandemic (OR 1.49, *p* < 0.001) and the QOL (OR 1.30, *p* < 0.001). Thus, the impact of the COVID-19 pandemic on mental health seems to be complex, demanding constant monitoring, especially given successive waves of the disease. We should note that, despite the social distancing, socialization was rated by Polish university students as being more satisfied with the housing situation (OR 0.81, *p* < 0.001) and relations with roommates (OR 0.88, *p* < 0.001).

Psychoactive substances influencing sleep include alcohol, cannabinoids, cocaine, or morphine [11,12,13,56,57]. The study confirmed the relationship between sleep and substance use. We showed that psychostimulants and sedatives-hypnotics were associated with AIS scores, while alcohol consumption was associated with lower AIS scores. However, the EPSS measurement showed that alcohol, cannabinoids, psychostimulants, and hypnotics affected daytime sleepiness. The finding of the effect of stimulants on sleep is not clear. For example, alcohol consumption in the initial stage may lead to a subjective improvement in sleep [58]. However, its only long-term use that can disturb sleep by extending its latency time, shortening the duration of sleep, and leading to frequent awakenings, thus significantly diminishing its quality [58,59]. The cannabinoids have a similar effect on sleep, but in the short-term manner, subjectively improving sleep quality [58]. In the study investigating the subjective evaluation of cannabis’ effect on sleep, the respondents indicated the ease of falling asleep as the main advantage of drug use [60]. Similar findings were confirmed in polysomnography studies of small THC doses, leading to shorter falling-asleep times, shorter sleep latencies, and shorter awakening times [61]. However, for chronic alcoholic use, even at low doses, there is a negative effect on sleep [62]. The most common subjective feelings are bizarre dreams, decreased sleep quality, and insomnia [63,64]. On the other hand, there is substantial evidence that substance use reduces the quality of sleep and adverse next-day effects on memory [65], as indicated in this study in a higher percentage of people with positive ESS and AIS scores. It is worth noting that the relationships between sleep and substance use seem to be bidirectional: the incidence of sleep disorders affects the increased risk of using psychoactive substances, and vice versa, their use leads to sleep deterioration. There is also evidence that the sleep disorders after discontinuation of psychostimulants may be an independent factor predisposed to relapse [62]. However, that requires in-depth analysis that goes beyond this study. The study showed that at the initial stage of the COVID-19 pandemic, there was a significant decrease in alcohol use among students in Poland. Similar findings were observed among students from the USA, Portugal, or Germany [66,67,68]. This effect was probably due to the prevailing restrictions, e.g., the closure of bars, restaurants, pubs, and universities, and the introduction of distance learning. This resulted in the deprivation of in-person social interactions often associated with alcohol consumption. This was also confirmed by the change in drinking habits, for instance, smaller amounts were consumed more often with the family [66,67,68]. The study among Spanish students showed a decline in alcohol and tobacco consumption as well as pro-health habits, such as increasing physical activity and consumption of fruit and vegetables. Some researchers hypothesized that the COVID-19 pandemic intensified some health-promoting habits due to the fear of the virus and death [25]. On the other hand, another study in the US assessing alcohol consumption before and after university closure showed an increased alcohol consumption and excessive searching for drinking opportunities among students [24]. Therefore, the patterns of substance use in the COVID-19 pandemic seem to vary given the study site, and its period and individual predispositions, consequently, it should be subjected to further monitoring. Interestingly, there was an inverse pattern in hypnotics/sedatives as their consumption increased during the COVID-19 pandemic. Undoubtedly, this effect may be explained by an increase in anxiety and depression, and sleep disorders [69]. Moreover, the period of the COVID-19 pandemic is also reflected in the assessment of our sleep. While the study results did not show any differences between the stages in the mean AIS values, these observations are visible in individual questions. The quality of sleep decreased significantly while its length increased.

The authors are aware of limitations of this study. The first limitation of the study was data collection. This resulted in difficulties in precise estimation of the number of participants who received the information and participants who replied. Additionally, it was not possible to determine the number of questionnaires that were initially finished and then not completed at each stage. Moreover, the anonymity of the study prevented the authors from providing information about the results and information about the potential need for specialist consultations. Another limitation was the limited representativeness of the Polish university students, such as most women, students of humanities, and first-year students. The measurements used also had some limitations. The self-reported scales were based on subjective feelings, but in-depth psychiatric research requires objectification, such as a psychiatric examination or standardized instruments. However, the scales used were comparable to the tools of, e.g., polysomnography, where satisfactory results were obtained [70,71]. The findings were also limited as no data on somatic diseases, medications, and mental disorders among the respondents and their family members were provided, as well as many other important sociodemographic factors. However, it should be emphasized that the study used reliable research methods and a large sample size. Limitations included the lack of measurements of the level of addiction to the substances used, as well as the assessment of day/night activity among respondents, which, according to the literature, have an impact on sleep quality [72].

To sum up, this study provides important findings on the epidemiology of sleep disorders, including somnolence and insomnia, and the subjective assessment of QOL among young adults studying in Poland. It indicated the specific psychoactive substance use patterns in Polish university students. It turned out that insomnia may be a common phenomenon as 54% of the surveyed students were prone to its symptoms. In addition, the study showed that the COVID-19 pandemic increased daytime sleepiness, worsening the quality of sleep without increasing the phenomenon of general insomnia. Compared to other reports, there was a significant increase in sleep problems among adults in Poland before the pandemic. However, the heterogeneity of the study groups, different periods of data collection, and different research methods prevented conclusive results from being obtained. Therefore, further research on sleep disorders requires an in-depth analysis based on a standardized methodology to objectify the final conclusions. Therefore, the authors have made an effort, and research is currently underway to disseminate the questionnaire in many countries around the world. The publication of the results is planned shortly.

## 5. Conclusions

Sleep disorders are a significant health problem for young adults living in Poland. The impact of the COVID-19 pandemic on sleep is not fully clear. As the pandemic has progressed, the quality of students’ sleep has decreased, and the phenomenon of daytime sleepiness may have also increased. There were no differences in severity of insomnia before and during the COVID-19 pandemic among Polish students. Ultimately, we found that substance use is widespread in young adults, affecting either sleep or their quality of life. Our findings also suggested the increased use of sedatives/hypnotics and decreased alcohol consumption during the COVID-19 pandemic.

## Figures and Tables

**Table 1:** Characteristics of the study group.

Variable		Overall Study Group N (%)	Before Pandemic N (%)	During Pandemic N (%)	Chi^2^	*p*
Sex	Female	11,985 (80.7)	6129 (79.2)	5859 (82.4)	25.38	<0.001
	Male	2859 (19.3)	1612 (20.8)	1248 (17.6)		
Age M ± SD		21.82 ± 2.8	21.97 ± 2.42	21.71 ± 4.74	-	<0.001
Study year	I	4842 (32.7)	1866 (24.1)	2977 (41.9)	573.05	<0.001
	II	2767 (18.6)	1717 (22.2)	1050 (14.8)		
	III	2781 (18.7)	1690 (21.8)	1093 (15.4)		
	IV	2080 (14.0)	1131 (14.6)	949 (13.4)		
	V	1984 (13.4)	1129 (14.6)	855 (12.0)		
	VI	390 (2.6)	208 (2.7)	183 (2.6)		
University profile	Medical	3425 (23.1)	2082 (26.9)	1344 (18.9)	133.09	<0.001
	Non-Medical	11,419 (76.9)	5659 (73.1)	5763 (81.1)		
Study course	Medical	3425 (23.1)	2082 (26.9)	1344 (18.9)	159.41	<0.001
	Technical	2676 (18.0)	1348 (17.5)	1329 (18.7)		
	Humanistic	5352 (36.1)	2524 (32.6)	2829 (39.8)		
	Biological	915 (6.1)	480 (6.2)	436 (6.1)		
	Economic	2472 (16.7)	1303 (16.8)	1169 (16.5)		
Faculty in the medical course	Medical/Dental	1884 (57.2)	1119 (56.0)	765 (58.9)	31.57	<0.001
	Pharmacy	354 (10.7)	263 (13.2)	91 (7.0)		
	Faculty of Health Sciences	1058 (32.1)	616 (30.8)	443 (34.1)		
COVID-19 pandemic status announcement	Before pandemic	7735 (52.1)	-	-	-	-
	During pandemic	7105 (47.9)	-	-		
**Use of stimulants in the last 3 months**						
Alcohol	Yes	13,039 (87.8)	7069 (91.3)	5973 (84.0)	183.55	<0.001
	No	1805 (12.2)	672 (8.7)	1134 (16.0)		
Cannabinoids	Yes	1978 (13.3)	1082 (14)	898 (12.6)	5.77	0.016
	No	12,866 (86.7)	6659 (86.0)	6209 (87.4)		
Psychostimulants	Yes	417 (2.9)	218 (2.8)	200 (2.8)	0.005	0.993
	No	14,427 (97.1)	7532 (97.2)	6907 (97.2)		
Sedatives	Yes	1933 (13.0)	904 (11.7)	1029 (14.5)	25.66	<0.001
	No	12,911 (87.0)	6837 (88.3)	6078 (85.5)		

**Table 2:** Linear regression model of the influence of sociodemographics and stimulants on AIS and ESS scales.

Explanatory Variable		Percentage of Positive Results	B	95%Cl	*t*	*p*
**Athens Insomnia Scale**						
COVID-19 pandemic status announcement	Before pandemic	54.83%	−0.085	[−0.174, 0.003]	−1.879	0.061
	During pandemic	54.51%	Ref.	Ref.	Ref.	Ref.
Sex	Female	55.90%	0.467	[0.378, 0.557]	10.283	**<0.001**
	Male	44.10%	Ref.	Ref.	Ref.	Ref.
Study year	I	55.48%	0.151	[0.004, 0.009]	2.100	**0.035**
	II	56.02%	0.182	[0.003, 0.015]	2.143	**0.015**
	III	53.90%	−0.042	[−0.208, 0.122]	−0.508	0.611
	IV	52.50%	−0.225	[−0.405, −0.043]	−2.434	**0.014**
	V	50.25%	−0.259	[−0.444, −0.075]	−2.759	**0.005**
	VI	54.99%	Ref.	Ref.	Ref.	Ref.
Study course	Medical	50.88%	−0.404	[−0.546, −0.263]	−5.586	**<0.001**
	Technical	52.90%	−0.194	[−0.345, −0.041]	−2.499	**0.012**
	Humanistic	57.91%	0.378	[0.253, 0.498]	6.022	**<0.001**
	Biological	60.15%	0.601	[0.358, 0.834]	5.052	**<0.001**
	Economic	49.80%	Ref.	Ref.	Ref.	Ref.
Faculty in the medical course	Medical/Dental	48.99%	−0.282	[−0.512, −0.052]	−2.401	**0.016**
	Pharmacy	47.74%	−0.137	[−0.491, 0.217]	−0.759	0.447
	Faculty of Health Sciences	54.86%	Ref.	Ref.	Ref.	Ref.
Alcohol	Yes	53.94%	−0.204	[−0.315, −0.094]	−3.63	**<0.001**
	No	55.70%	Ref.	Ref.	Ref.	Ref.
Cannabinoids	Yes	53.48%	Ref.	Ref.	Ref.	Ref.
	No	54.26%	−0.031	[−0.134, 0.072]	−0.591	0.553
Psychostimulants	Yes	58.85%	Ref.	Ref.	Ref.	Ref.
	No	54.02%	−0.356	[−0.568, −0.144]	−3.291	**<0.001**
Sedatives	Yes	78.43%	Ref.	Ref.	Ref.	Ref.
	No	50.52%	−1.700	[−1.800, −1.599]	−33.115	**<0.001**
**Epworth Sleepiness Scale**						
COVID-19 pandemic status announcement	Before pandemic	28.33%	0.326	[0.241, 0.412]	7.493	**<0.001**
	During pandemic	23.62%	Ref.	Ref.	Ref.	Ref.
Sex	Female	27.48%	0.477	[0.390, 0.562]	10.915	**<0.001**
	Male	20.21%	Ref.	Ref.	Ref.	Ref.
Study year	I	26.68%	0.201	[0.066, 0.336]	2.923	**0.003**
	II	27.25%	0.223	[0.063, 0.383]	2.732	**0.006**
	III	26.70%	0.055	[−0.103, 0.215]	0.685	**0.049**
	IV	23.89%	−0.151	[−0.324, 0.022]	−1.701	0.088
	V	24.19%	−0.167	[−0.344, 0.010]	−1.847	0.065
	VI	27.11%	Ref.	Ref.	Ref.	Ref.
Study course	Medical	30.24%	0.409	[0.272, 0.545]	5.867	**<0.001**
	Technical	24.80%	−0.086	[−0.232, 0.060]	−1.154	0.249
	Humanistic	25.11%	−0.033	[−0.151, 0.085]	−0.5514	0.581
	Biological	27.18%	−0.044	[−0.268, 0.180]	−0.384	0.701
	Economic	23.42%	Ref.	Ref.	Ref.	Ref.
Faculty in the medical course	Medical/Dental	28.82%	−0.268	[−0.500, −0.035]	−2.261	**0.023**
	Pharmacy	31.92%	0.081	[−0.2767, 0.439]	0.446	0.655
	Faculty of Health Sciences	32.20%	Ref.	Ref.	Ref.	Ref.
Alcohol	Yes	26.32%	0.196	[0.090, 0.302]	3.626	**<0.001**
	No	24.31%	Ref.	Ref.	Ref.	Ref.
Cannabinoids	Yes	28.89%	Ref.	Ref.	Ref.	Ref.
	No	25.65%	−0.147	[0.048, 0.247]	−2.916	**0.003**
Psychostimulants	Yes	32.30%	Ref.	Ref.	Ref.	Ref.
	No	25.90%	−0.242	[−0.455, −0.048]	−2.427	**0.015**
Sedatives	Yes	30.26%	Ref.	Ref.	Ref.	Ref.
	No	25.45%	−0.143	[−0.243, −0.043]	−2.809	**<0.001**

Notes: Statistically significant values are in bold with the significance level set at *p* < 0.05. Abbreviations: B—coefficient value of a given variable, 95% CI—confidence interval of B coefficient, *t*—test value, *p*—statistical significance. M—mean, SD—standard deviation.

**Table 3:** Influence of sociodemographic variables, pandemic, stage of studies, insomnia, and daytime sleepiness on subjective QOL assessment in a linear regression model.

Explanatory Variable		B	95%CI	*t*	*p*
**MANSA Scale**					
COVID-19 pandemic status announcement	Before pandemic	0.430	[0.199, –0.661]	3.653	**<0.001**
	During pandemic	Ref.	Ref.	Ref.	Ref.
Sex	Female	−0.237	[−0.468, −0.006]	−2.015	**0.043**
	Male	Ref.	Ref.	Ref.	Ref.
Study year	I	−0.866	[−1.223, −0.504]	−4.682	**<0.001**
	II	−0.545	[−0.975, −0.115]	−2.489	**0.001**
	III	−0.644	[−1.072, −0.216]	−2.953	**0.003**
	IV	0.385	[−0.08, 0.851]	1.616	**0.105**
	V	0.704	[0.227, 1.181]	2.896	**0.003**
	VI	Ref.	Ref.	Ref.	Ref.
Study course	Medical	2.238	[1.873, 2.602]	12.024	**<0.001**
	Technical	0.049	[−0.341, 0.439]	0.248	**0.804**
	Humanistic	−1.503	[−1.817, −1.188]	−9.360	**<0.001**
	Biological	−0.891	[−1.491, −0.291]	−2.912	**0.003**
	Economic	Ref.	Ref.	Ref.	Ref.
Faculty in the medical course	Medical/Dental	1.401	[0.821, 1.982]	4.731	**<0.001**
	Pharmacy	−0.326	[−1.221, 0.569]	−0.713	0.476
	Faculty of Health Sciences	Ref.	Ref.	Ref.	Ref.
Insomnia (AIS)	Yes	−8.923	[−9.255, −8.589]	−52.465	**<0.001**
	No	Ref.	Ref.	Ref.	Ref.
Daytime sleepiness (ESS)	Yes	−3.297	[−3.706, −2.888]	−15.801	**<0.001**
	No	Ref.	Ref.	Ref.	Ref.
Alcohol	Yes	0.552	[0.267, 0.834]	3.793	**<0.001**
	No	Ref.	Ref.	Ref.	Ref.
Cannabinoids	Yes	Ref.	Ref.	Ref.	Ref.
	No	−0.587	[−.0853, −0.320]	−4.301	**<0.001**
Psychostimulants	Yes	Ref.	Ref.	Ref.	Ref.
	No	2.056	[1.501, 2.603]	7.372	**<0.001**
Sedatives	Yes	Ref.	Ref.	Ref.	Ref.
	No	3.198	[2.934, 3.465]	23.719	**<0.001**

Notes: Statistically significant values are in bold with the significance level set at *p* < 0.05. Abbreviations: B—coefficient value of a given variable, 95%CI—confidence interval of B coefficient, *t*—test value, *p*—statistical significance.

**Table 4:** Polynomial logistic regression analysis of the relationship between separate MANSA subscales and the COVID-19 pandemic in the ordinance.

Question	Mean Score for Both Phases of the Study	Before the Pandemic Announcement	
	M (SD)	OR (95%CI)	*p*
How satisfied are you with your life as a whole today?	4.592 (1.34)	1.30 (1.22–1.38)	**<0.001**
How satisfied are you with your job (or sheltered employment, or training/education as your main occupation)?	4.441 (1.34)	0.915 (0.86–0.97)	**<0.001**
How satisfied are you with your financial situation?	4.106 (1.53)	1.11 (1.04–1.18)	**0.001**
How satisfied are you with the number and quality of your friendships?	4.667 (1.72)	1.09 (1.02–1.16)	**0.006**
How satisfied are you with your leisure activities (hobby)?	4.223 (1.66)	1.04 (0.97–1.11)	0.201
How satisfied are you with your accommodation?	4.764 (1.60)	0.81 (0.77–0.87)	**<0.001**
How satisfied are you with your personal safety?	5.165 (1.42)	1.12 (1.06–1.20)	**<0.001**
How satisfied are you with the people that you live with?	5.049 (1.55)	0.88 (0.83–0.94)	**<0.001**
How satisfied are you with your sexual life?	4.268 (1.93)	1.09 (1.03–1.17)	**0.002**
How satisfied are you with your relationship with your family?	4.962 (1.44)	1.14 (1.07–1.22)	**<0.001**
How satisfied are you with your physical health?	4.301 (1.48)	1.04 (0.98–1.10)	0.216
How satisfied are you with your mental health?	4.128 (1.68)	1.49 (1.40–1.58)	**<0.001**

Notes: Statistically significant values are in bold with the significance level set at *p* < 0.05. OR—odds ratio.

**Table 5:** Polynomial logistic regression analysis of sociodemographics and pandemic effects on the frequency of stimulants/alcohol and drug use.

Variables		Alcohol		Cannabinoids		Psychostimulants		Sedatives	
		OR (Cl 95%)	*p*	OR (Cl 95%)	*p*	OR (Cl 95%)	*p*	OR (Cl 95%)	*p*
Sex (Female—Ref.)	Male	1.45 (1.30–1.63)	**<0.001**	2.10 (1.79–2.46)	**<0.001**	2.10 (1.55–2.87)	**<0.001**	0.66 (0.54–0.81)	**<0.001**
Study year (I year—Ref.)	II	0.83 (0.73–0.94)	**0.002**	0.85 (0.68–1.05)	0.147	0.81 (0.52–1.27)	0.362	1.04 (0.85–1.27)	0.674
	III	1.02 (0.90–1.16)	0.701	1.04 (0.85–1.26)	0.733	0.99 (0.66–1.49)	0.976	1.02 (0.84–1.24)	0.811
	IV	1.05 (0.92–1.21)	0.401	0.87 (0.70–1.09)	0.238	093 (0.60–1.45)	0.760	0.83 (0.67–1.03)	0.096
	V	1.13 (0.99–1.30)	0.071	0.69 (0.54–0.89)	**<0.001**	0.61 (0.36–1.04)	0.720	0.84 (0.67–1.06)	0.139
	VI	1.36 (1.04–1.80)	**0.027**	0.61 (0.36–1.03)	**0.050**	0.90 (0.36–2.25)	0.826	1.04 (0.68–1.58)	0.855
Study course (Biological—Ref.)	Economic	1.00 (0.82–1.22)	0.981	0.88 (0.61–1.28)	0.156	0.79 (0.39–1.59)	0.527	1.01 (0.73–1.40)	0.913
	Humanistic	0.83 (0.69–1.00)	**0.050**	0.79 (0.58–1.09)	0.148	1.06 (0.58–1.97)	0.835	1.40 (1.04–1.87)	**0.029**
	Medical	0.88 (0.73–1.08)	0.226	0.74 (0.54–1.01)	0.059	0.94 (0.48–1.83)	0.861	0.94 (0.68–1.29)	0.710
	Technical	1.18 (0.97–1.45)	0.085	0.96 (0.70–1.30)	0.799	1.12 (0.58–2.15)	0.732	0.64 (0.46–0.88)	**<0.001**
Faculty in the medical course (Pharmaceutical—Ref.)	Medical and dental	1.57 (1.06–2.32)	**0.025**	1.65 (0.78–3.52)	0.187	3.05 (0.41–22.79)	0.276	0.78 (0.43–1.43)	0.430
	Health sciences	1.44 (0.96–2.17)	0.074	0.85 (0.38–1.90)	0.700	1.24 (0.14–10.47)	0.839	0.788 (0.42–1.49)	0.463
COVID-19 pandemic status announcement (During pandemic—Ref.)	Before pandemic	1.35 (1.27–1.44)	**<0.001**	1.10 (0.98–1.23)	0.083	0.93 (0.73–1.18)	0.571	0.76 (0.69–0.85)	**<0.001**

Notes: Statistically significant values are in bold with the significance level set at *p* < 0.05.

## Data Availability

The data presented in this study are available on request from the corresponding author.

## Supplementary Materials

The following supporting information can be downloaded at: https://www.mdpi.com/article/10.3390/jcm11082106/s1. Table S1. A detailed list of the individual questions included in the Athens Insomnia Scale, divided into the period before and during the COVID-19 pandemic. Table S2. A detailed list of questions included in the Epworth Sleepiness Scale, divided into the period before and during the COVID-19 pandemic.
