# Supplementary material for: Assessment of Insomnia Symptoms, Quality of Life, Daytime Sleepiness, and Psychoactive Substance Use among Polish Students: A Cross-Sectional Online Survey for Years 2016–2021 before and during COVID-19 Pandemic
Source: J Clin Med. 2022 Apr 9;11(8):2106. doi: 10.3390/jcm11082106 (PMC9026410; doi:10.3390/jcm11082106)
Supplement: Supplementary file 1 [file jcm-11-02106-s001.zip › Supplementary materials.pdf]

Table S1. A detailed list of the individual questions included in the Athens Insomnia Scale, divided into the period before and during the COVID-19 pandemic.

Sleep factors		Whole group N (%)	Before the COVID-19 pandemic N (%)	COVID-19 pandemic N (%)	P	Cramér's V
Sleep induction	No problem	3594 (24.2)	2111 (27.3)	1483 (20.9)	<0.001	0.078
	Slightly delayed	4595(30.9)	2369 (30.6)	2226 (31.3)		
	Markedly delayed	4762 (32.1)	2319 (30.0)	2443 (34.4)		
	Very delayed or did not sleep at all	1897 (12.8)	942 (12.1)	995 (13.4)		
Awakenings during the night	No problem	2573 (17.3)	1349 (17.4)	1224 (17.2)	0.010	0.020
	Minor problem	8279 (55.8)	4370 (56.4)	3909 (55.0)		
	Considerable problem	3416 (23.0)	1739 (22.5)	1677 (23.6)		
	Serious problem or did not sleep at all	580 (3.9)	283 (3.7)	297 (4.2)		
Final awakening	Not earlier	7084 (47.7)	3861 (49.9)	3223 (45.4)	<0.001	0.052
	A little earlier	5581 (37.6)	2853 (36.9)	2728 (38.4)		
	Markedly earlier	1749 (11.8)	831 (10.7)	918 (12.9)		
	Much earlier or did not sleep at all	434 (2.9)	196 (2.5)	238 (3.3)		
Total sleep duration	Sufficient	3644 (24.5)	1623 (21.0)	2021 (28.4)	<0.001	0.101
	Slightly insufficient	6390 (43.1)	3338 (43.1)	3052 (42.9)		
	Markedly insufficient	4278 (28.8)	2503 (32.3)	1775 (25.0)		
	Very insufficient or did not sleep at all	536 (3.6)	277 (3.6)	259 (3.6)		
Sleep quality	Satisfactory	4120 (27.7)	2329 (30.1)	1791 (25.1)	<0.001	0.056
	Slightly unsatisfactory	6601 (44.5)	3371 (43.6)	3230 (45.5)		
	Markedly unsatisfactory	3396 (22.9)	1685 (21.8)	1711 (24.1)		
	Very unsatisfactory or did not sleep at all	731 (4.9)	356 (4.5)	375 (5.3)		
Well-being during the day	Normal	5817 (39.2)	3055 (39.5)	2762 (38.9)	0.331	0.015
	Slightly decreased	5938 (40.0)	3119 (40.3)	2819 (39.7)		
	Markedly decreased	2689 (18.1)	1364 (17.6)	1325 (18.6)		
	Very decreased	404 (2.7)	203 (2.5)	201 (2.8)		
Functioning capacity during the day	Normal	6435 (43.4)	3405 (44.0)	3030 (42.6)	0.133	0.019
	Slightly decreased	6301 (42.4)	3275 (42.2)	3026 (42.6)		
	Markedly decreased	1840 (12.4)	917 (11.9)	923 (13.0)		
	Very decreased	272 (1.8)	144 (1.9)	128 (1.8)		
Sleepiness during the day	None	955 (6.4)	412 (5.4)	543 (7.6)	<0.001	0.061
	Mild	6940 (46.7)	3525 (45.5)	3415 (48.1)		
	Considerable	5598 (37.7)	3068 (39.6)	2530 (35.6)		
	Intense	1355 (9.2)	736 (9.5)	619 (8.7)		

Table S2. A detailed list of questions included in the Epworth Sleepiness Scale, divided into the period before and during the COVID-19 pandemic.

**How likely are you to doze off or fall asleep in the following situations?**		**Whole group N (%)**	**Before the COVID-19 pandemic N (%)**	**COVID-19 pandemic N (%)**	**P**	**Cramér's V**
Sitting and reading	No chance of dozing	5168 (34.8)	2520 (32.6)	2648 (37.2)	<0.001	0.065
	Slight chance of dozing	4427 (29.8)	2269 (29.3)	2158 (30.4)		
	Moderate chance of dozing	3519 (23.7)	1944 (25.1)	1575 (22.2)		
	High chance of dozing	1734 (11.7)	1008 (13.0)	726 (10.2)		
Watching TV ·	No chance of dozing	4057 (27.3)	2023 (26.1)	2034 (28.6)	0.001	0.032
	Slight chance of dozing	4628 (31.2)	2420 (31.3)	2208 (31.1)		
	Moderate chance of dozing	3963 (26.7)	2090 (27.0)	1873 (26.4)		
	High chance of dozing	2200 (14.8)	1208 (15.6)	992 (13.9)		
Sitting inactive in a public place (e.g., a theater or a meeting)	No chance of dozing	9522 (64.1)	4786 (61.8)	4736 (66.6)	<0.001	0.053
	Slight chance of dozing	3462 (23.3)	1891 (24.4)	1571 (22.1)		
	Moderate chance of dozing	1387 (9.3)	805 (10.4)	582 (8.2)		
	High chance of dozing	477 (3.3)	259 (3.4)	218 (3.1)		
As a passenger in a car for an hour without a break	No chance of dozing	3915 (26.4)	1901 (24.6)	2014 (28.3)	<0.001	0.057
	Slight chance of dozing	4246 (28.6)	2181 (28.2)	2065 (29.1)		
	Moderate chance of dozing	3804 (25.6)	2016 (26.0)	1788 (25.2)		
	High chance of dozing	2883 (19.4)	1643 (21.2)	1240 (17.4)		
Lying down to rest in the afternoon when circumstances permit	No chance of dozing	1322 (8.9)	631 (8.1)	691 (9.7)	<0.001	0.078
	Slight chance of dozing	2759 (18.6)	131 (16.9)	1449 (20.4)		
	Moderate chance of dozing	4248 (28.6)	2119 (27.4)	2129 (30.0)		
	High chance of dozing	6519 (43.9)	3681 (47.6)	2838 (29.9)		
Sitting and talking to someone	No chance of dozing	13072 (88.0)	6804 (87.9)	6268 (88.2)	0.355	0.014
	Slight chance of dozing	1545 (10.4)	825 (10.7)	720 (10.2)		
	Moderate chance of dozing	190 (1.3)	95 (1.2)	95 (1.3)		
	High chance of dozing	41 (0.3)	17 (0.2)	24 (0.3)		
Sitting quietly after a lunch without alcohol	No chance of dozing	4453 (30.0)	2263 (29.2)	2190 (30.8)	0.076	0.021
	Slight chance of dozing	5055 (34.0)	2646 (34.2)	2409 (33.9)		
	Moderate chance of dozing	3592 (24.2)	1881 (24.3)	1711 (24.1)		
	High chance of dozing	1748 (11.8)	951 (12.3)	797 (11.2)		
In a car, while stopped for a few minutes in traffic	No chance of dozing	13991 (94.2)	7269 (93.9)	6722 (94.6)	0.113	0.020
	Slight chance of dozing	715 (4.8)	400 (5.2)	315 (4.4)		
	Moderate chance of dozing	102 (0.7)	55 (0.7)	47 (0.7)		
	High chance of dozing	40 (0.3)	17 (0.2)	23 (0.3)		
